# New Two-Dimensional Materials Obtained by Functionalization of Boron Graphdiyne Layers with Nickel

**DOI:** 10.3390/nano14211706

**Published:** 2024-10-25

**Authors:** Estefanía Germán, María J. López, Julio A. Alonso

**Affiliations:** 1 Departamento de Física Teórica, Atómica y Optica, University of Valladolid, 47011 Valladolid, Spain; mariajlopez@uva.es (M.J.L.); jaalonso@uva.es (J.A.A.); 2 Donostia International Physics Center (DIPC), 20018 San Sebastián, Spain

**Keywords:** DFT, electronic structure, nickel, carbon-based materials, semiconductor

## Abstract

The decoration of hexagonal boron graphdiyne (BGDY) layers with Ni atoms has been investigated by density functional calculations. For one, two, and three Ni atoms per hexagon, the BGDY structure is approximately maintained. Decoration with six Ni atoms per hexagon leads to the formation of a novel, very stable two-dimensional material in which the hexagonal structure of BGDY is substantially distorted. The Ni-doped materials have a semiconductor character, and the electronic band gap width can be tailored by varying the amount of adsorbed Ni. BGDY-2Ni, BGDY-3Ni, and BGDY-6Ni have electronic band gaps promising for infrared detectors. This work shows that computer simulation helps to discover new materials by the functionalization of layered carbon materials with metal atoms.

## 1. Introduction

One of the objectives of materials science is to discover new materials with novel structures, properties, and applications. This is the case, in particular, for two-dimensional (2D) materials. The search is performed by utilizing experimental methods, theoretical methods, computer simulation techniques, or a combination of these tools. After the experimental isolation of a monolayer and a few layers of graphene [1], a lot of work has been performed on layered materials, and exotic properties and new applications have been discovered [2,3,4]. Graphene has catalyzed the research on 2D carbon materials. Two new 2D carbon materials are graphyne and graphdiyne (GDY), with a layer structure formed by benzenic rings connected by carbon chains containing diacetylenic linkages. The difference between graphyne and GDY is the length of the carbon chains. GDY has been synthesized on the surface of copper [5]. Graphyne has been synthesized in bulk phase, and the interlayer stacking was investigated [6]. Single layer graphyne has also been studied by theoretical calculations [7,8]. The graphyne and GDY layers present a uniform arrangement of triangular holes, and this arrangement leads to electronic and optical properties showing interesting differences with respect to graphene, such as a band gap opening [9]. Other properties found in studies of GDY are a high third-order nonlinear optical susceptibility [10] and high fluorescence efficiency [11].

The properties of layered carbon materials can be modified by doping and functionalization with other chemical species. Graphane is a phase, with stoichiometry CH, obtained by saturating graphene with chemisorbed hydrogen atoms. The possibility of forming graphane was predicted by theoretical calculations [12,13], and the material was synthesized in the laboratory [14,15,16] a few years later. Each atom of the carbon layer is covalently bonded to an H atom in graphane, and the H atoms are bonded to C atoms on both sides of the layer in an alternating manner. A band gap of 5.2 eV has been predicted for graphane [17]. The deposition of metal atoms and nanoparticles on GDY has been studied in several works [18,19,20,21].

**Figure 1:**
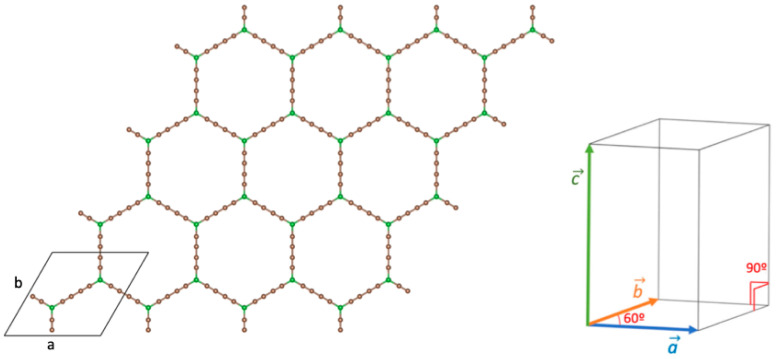
The honeycomb structure of boron–graphdiyne and the three-dimensional supercell: *a* = *b* and γ (angle between $\vec{a}$ and $\vec{b}$ ) = 60°. Boron and carbon atoms are represented by green and brown spheres, respectively. In the calculations for doped BGDY, the constraints on *a*, *b,* and γ have been relaxed.

A new member of the GDY family is boron–graphdiyne (BGDY). In this layered structure, a hexagonal honeycomb lattice is formed with boron atoms interconnected by carbon butadiyne chains. The structure has large holes, as shown in Figure 1. BGDY has been synthesized by a bottom-up strategy [22]. BGDY is a semiconductor with high electron and hole mobilities [23,24]. The empty hollows in the planar structure make GDY and BGDY potentially useful materials for filtration [25,26] and storage purposes. The sodium-storage capacity of BGDY has been investigated [22], and, in general, so has its application as an anode for alkaline and alkaline–earth ion batteries [24,27]. Hussain et al. [28] explored the hydrogen storage properties of BGDY nano-sheets functionalized with light metal atoms (Li, Na, K, Ca). By performing density functional (DFT) calculations [29], we have investigated the adsorption of V, Co, and Pd atoms and small clusters of those elements on BGDY [30]. The adsorption energies are sufficiently high to stabilize those metal clusters on the BGDY substrate, and this is promising for hydrogen storage, which is known to be enhanced by functionalizing the carbonaceous substrates with metal atoms and nanoparticles [31,32,33,34]. The binding energies of V, Co, and Pd atoms on BGDY [30] make these systems stable and promising for single-atom catalysis [35].

Modifying BGDY by functionalization may lead to new materials with novel properties. Motivated by previous research [22,24,27,28,30,34], we present a study of the functionalization of BGDY with Ni by placing the Ni atoms at the most stable adsorption positions of the BGDY layer. The functionalization induces rearrangements of the BGDY structure, which is sensitive to the number of Ni atoms adsorbed. New materials with interesting structures are predicted. In particular, an intriguing 2D structure with six Ni atoms decorating each hexagon in BGDY, which we call BGDY-6Ni, has been discovered. The electronic properties of the Ni-doped BGDY materials have been studied.

## 2. Theoretical Method and Adsorption Model

The structures of BGDY functionalized with Ni atoms have been investigated using the DFT [29] formalism, implemented in the quantum-ESPRESSO code, version 6.4.1 [36]. Electronic exchange-correlation effects have been taken into account with the Perdew–Burke–Ernzerhof generalized gradient approximation functional, GGA-PBE [37,38]. Interactions between the electrons and the ion cores were modeled using the projected augmented wave method (PAW) [39,40]; namely, the C.pbe-n-kjpaw_psl.1.0.0.UPF pseudopotential for carbon, B.pbe-n-kjpaw_psl.1.0.0.UPF for boron, and Ni.pbe-n-kjpaw_psl.0.1.UPF for nickel. These pseudopotentials are available from the quantum ESPRESSO website [41] and correspond to the following electronic configurations: 2*s*^2^ 2*p*^1^ for B, 2*s*^2^ 2*p*^2^ for C, and 4*s*^2^ 3*d*^8^ for Ni. This choice amounts to considering 4 external electrons for C, 3 for B, and 10 for Ni. An energy cutoff of 45 Ry was selected for the plane wave basis used to expand the Kohn–Sham orbitals and 350 Ry for the charge density. These cutoffs led to well-converged results. The Brillouin zone was sampled with a 3 × 3 × 1 Monkhorst–Pack *k*-point grid [42]. The Grimme-D3 method was used to include dispersion effects [43]. A value of 0.0002 Ry was selected as the criterion for energy convergence in the structural optimizations, and it was required that the forces acting on each atom be below 0.001 Ry/Å. All the calculations were carried out in spin-polarized mode.

The structure of the BGDY substrate is shown in Figure 1. It is a honeycomb structure with the boron atoms at the vertices of the hexagons, linked by butadiyne chains. In the calculations, the BGDY layer was modeled using a repetitive supercell, sketched in Figure 1, containing 14 atoms: twelve C atoms and two B atoms. The length of the cell in the direction perpendicular to the layer is 20 Å, large enough to avoid interactions between periodic images of the layer. The optimized lattice constants are *a* = *b* = 11.88 Å. The B–C bond length is *d*(B–C) = 1.52 Å, and the bond lengths of triple and single carbon–carbon bonds are *d*(C≡C) = 1.24 Å and *d*(C–C) = 1.35 Å, respectively, in agreement with previous works [23,30]. The decoration of BGDY with one, two, three, and six Ni atoms per cell has been investigated. In these cases, the lattice constants *a* and *b*, as well as the angle γ between the two lattice vectors of the unit cell (see Figure 1), were allowed to vary in order to minimize the total energy of the system. This led to distortions in the structure of the BGDY substrate. To study the electronic properties, the band structure and the density of electronic states (DOSs) have been calculated, and, for this purpose, calculations at fixed geometry were performed, employing 20 × 20 × 1 *k*-point grids.

The thermal stability of BGDY-6Ni has been investigated by performing ab initio molecular dynamics simulations, AIMD. The simulations were performed for three different repetitive supercells, formed by 2 × 2, 3 × 2, and 4 × 3 unit cells, that contain a total of 80, 120, and 240 atoms, respectively. Calculations at the Gamma point of the reciprocal lattice yield good accuracy for these relatively large supercells. Constant temperature (within the NVT canonical ensemble) MD simulations were performed using the Andersen thermostat [44] at three temperatures: T = 500 K, 1000 K, and 1500 K. The atomic velocities are scaled at every time step according to the simulation temperature. A time step of 1.94 fs has been used, and simulation times have been extended up to 12 ps.

## 3. Adsorption of One Ni Atom Per Cell

**Figure 2:**
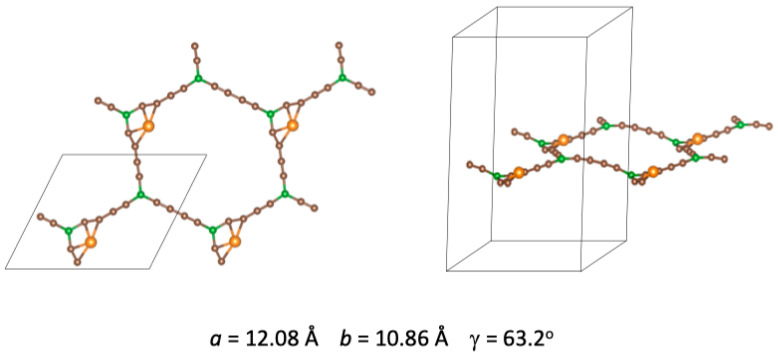
Top and side views of the preferential adsorption geometry for a single Ni atom per cell of BGDY. Relaxation of the cell size and shape (*a*, *b*, and γ ) is allowed in the calculations. The Ni atoms are represented by the spheres in orange color.

When one Ni atom is adsorbed per cell, the most stable adsorption sites are the corners of the hexagonal holes [30]. Figure 2 shows the top and side views of the optimized lowest energy structure. Four unit cells are included to appreciate the structural distortion with respect to pristine BGDY. The Ni atoms induce small distortions in the hexagons of the BGDY layer: the angle γ = 63.2° is different from γ = 60° of pristine BGDY, and the lattice constants are *a* = 12.08 Å, *b* = 10.86 Å, showing that the value of *a* is a bit larger than the value *a* = 11.88 Å of pristine BGDY. A small buckling of the layer, that is, a deviation from planarity, can be appreciated.

The adsorption energy of the Ni atom, defined in Equation (1), is given in Table 1. *E*(BGDY) and *E*(BGDY-1Ni) are the energies per cell of pristine BGDY and BGDY with one Ni atom adsorbed, respectively, and *E*(Ni) is the energy of the free Ni atom. The positive value of *E_ads_*(first Ni atom) means that the process is exothermic, and the strength of the binding, 3.37 eV, is sizeable. The distortion of the BGDY layer, in this case, and for a higher Ni concentration (to be discussed next), helps to provide optimal accommodation for the adsorbed Ni atoms; that is, the energy toll paid by the distortion becomes more than compensated for the increase in the adsorption energy of the Ni atoms in the optimized environment.

*E_ads_*(1st Ni atom) = *E*(BGDY) + *E*(Ni) − *E*(BGDY-1Ni) (1)

**Figure 3:**
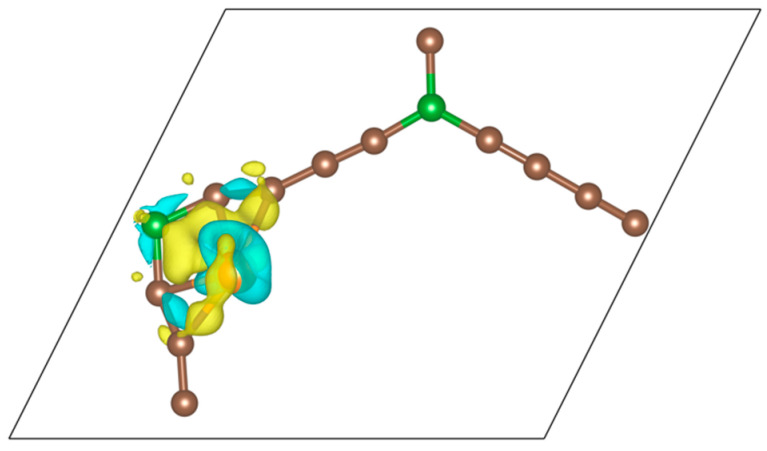
Electron density redistribution Δ ρ = ρ (BGDY-1Ni) − ρ (BGDY) − ρ (Ni), occurring after adsorption of the Ni atom. The density increases in the yellow regions at the expense of a decrease in the blue regions. The plotted surfaces correspond to Δ ρ = ± 0.007 e/Å^3^.

Figure 3 shows the electron density redistribution that occurs after the adsorption of the Ni atom. The density increases (yellow surfaces) in the regions between the Ni atom and its C and B neighbors. Two Ni–C covalent bonds can be identified and also a four-center bond involving Ni, B, and two C atoms. The structure in Figure 2, with the Ni atoms well separated in space, could be of interest for single-atom catalysis.

## 4. Adsorption of Two Ni Atoms per Cell

**Figure 4:**
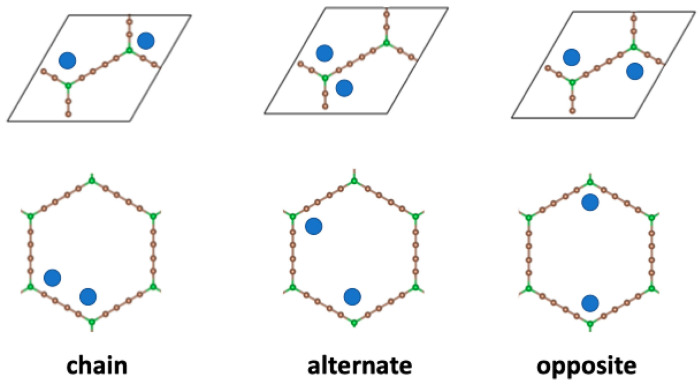
Upper panels: inequivalent initial configurations for the adsorption of two Ni atoms per unit cell. Bottom panels: the same configurations showing the positions of the metal Ni (chain, alternate, opposite) in the hexagons forming the structure of BGDY.

**Figure 5:**
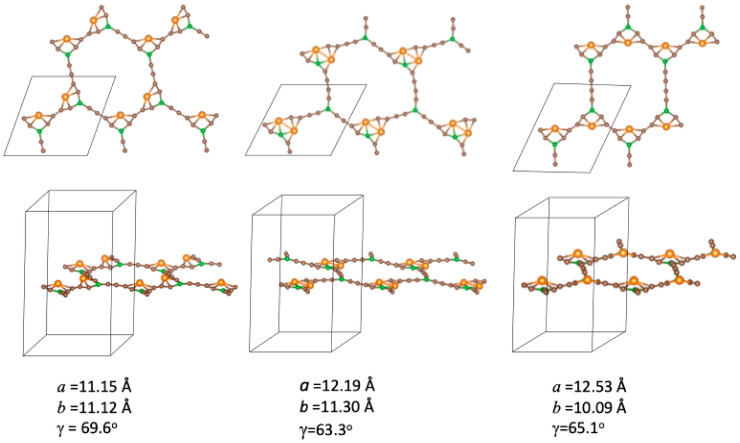
Top and side views of the optimized structures of BGDY functionalized with two Ni atoms per cell in chain, alternate, and opposite configurations.

When two metal atoms are adsorbed per unit cell, three non-equivalent initial configurations exist, with the atoms decorating different corners of the BGDY structure; these are shown in Figure 4. Those configurations correspond to two atoms inside each hexagon in opposite, adjacent (chain configuration), or alternate vertices, respectively. Starting with those initial configurations, the final configurations obtained after full structural optimization, allowing for relaxation of *a*, *b*, and γ, are displayed in Figure 5. In the three cases, the deviations of the BGDY layer from planarity are quite small (smaller compared to the case of one Ni atom per cell), although the Ni atoms protrude a bit from the BGDY plane. Also, in-plane distortions occur, and the value of γ increases.

The energies corresponding to the three final configurations are compared in Table 2. The lowest energy structure arises from the alternate configuration of the Ni atoms in each hexagon, which is energetically more stable than the other two configurations by amounts close to 1 eV per cell. There is an inverse correlation between stability and the deviation of γ from γ = 60°; larger deviations lead to lower stability. An additional factor enhancing the stability of the alternate configuration is the formation of bonds between Ni atoms of adjacent hexagons at the expense of breaking one B–C bond. In this configuration, the structure of the atomic environment around the B atoms is perturbed by the presence of Ni atoms, and, since this affects three alternate B atoms of each hexagon, the hexagons deform. The Ni-B bond distances are 1.95 and 1.98 Å.

The adsorption energy for the addition of the second Ni atom is calculated as follows:

*E_ads_*(2nd Ni atom) = *E*(BGDY-1Ni) + *E*(Ni) − *E*(BGDY-2Ni), (2)

where the energies appearing in Equation (2) have already been defined, except *E*(BGDY-2Ni), which is the energy per cell of BGDY with two Ni atoms adsorbed. These adsorption energies are given in Table 2 for the three configurations of the Ni atoms. The adsorption energy of the second metal atom in the most favorable configuration, the alternate one, is compared to that of the first Ni atom in Table 1. The adsorption energy of the second Ni atom is larger, and the reason is the formation of Ni–Ni bonds, with bond lengths of *d*(Ni–Ni) = 2.58 Å, a bit larger than the nearest neighbor distance of 2.49 Å in bulk nickel [45]. Due to the formation of Ni–Ni bonds, each Ni atom has five neighbors. By comparing with the case of a single Ni atom per unit cell, this reveals the adaptability of Ni to environments with different numbers of neighbors, being able to form covalent, metallic, and mixed metallic–covalent bonds. The same effect will be seen for higher Ni concentrations.

## 5. Adsorption of Three Ni Atoms Per Cell

**Figure 6:**
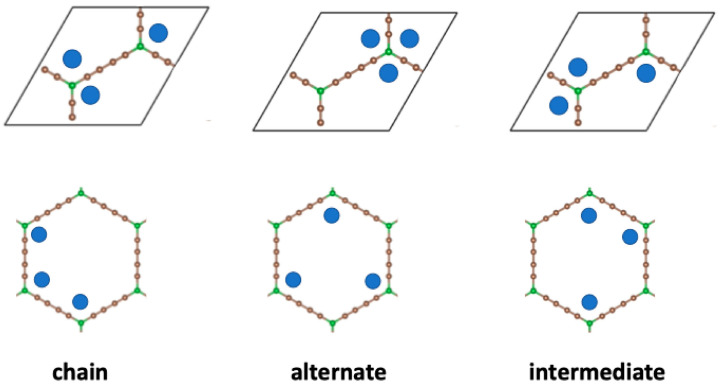
Upper panels: nonequivalent initial decorating configurations for the adsorption of three Ni atoms per unit cell. Bottom panels: the same configurations showing the positions of the Ni atoms (chain, alternate, intermediate) in the hexagons forming the structure of BGDY.

**Figure 7:**
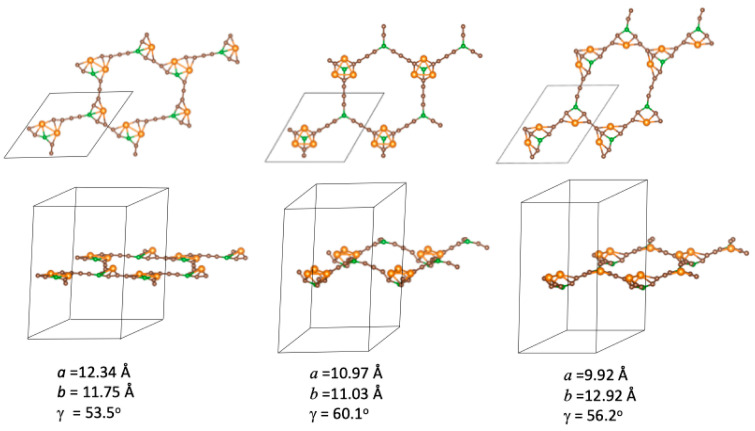
Top and side views of the optimized structures of BGDY functionalized with three Ni atoms per cell in chain (**left**), alternate (**middle**), and intermediate (**right**) configurations.

When three Ni atoms are adsorbed per unit cell, there are three non-equivalent initial configurations, shown in Figure 6, with the Ni atoms decorating different corners of the BGDY structure. Those structures correspond to three Ni atoms inside each hexagon, in positions in which (a) the Ni atoms form a chain, (b) the Ni atoms occupy alternate vertices, and (c) the Ni atoms are in an intermediate configuration. Starting with those initial configurations, the structures found after structural optimization allowing for distortion of the shape and size of the unit cell are shown in Figure 7. The optimized chain configuration exhibits substantial in-plane distortions, with γ = 53.5°, but there is practically no deviation of the BGDY layer from planarity. The Ni atoms form bonds with B atoms. Also, two Ni atoms of each hexagon form Ni–Ni bonds of length *d*(Ni–Ni) = 2.57 Å, with Ni atoms of adjacent hexagons. In contrast, in the other two configurations, the deviations of γ from the starting value are smaller, but the structures show buckling. Interestingly, the metal atoms form triangular trimers above alternate B atoms in the optimized alternate configuration. Those triangles are near-equilateral, with bond lengths *d*(Ni–Ni) = 2.53–2.54 Å. The covered B atoms are below the original BGDY plane, and the uncovered B atoms are above.

The lowest energy structure arises from the chain configuration, and the relative energies that correspond to the three optimized configurations are given in Table 3. The most stable structure results from a combination of two effects: the preservation of the planarity of the BGDY layer and the formation of bonds of Ni atoms with B and C atoms. The adsorption energy of the third Ni atom is defined as follows:

*E_ads_*(3rd Ni atom) = *E*(BGDY-2Ni) + *E*(Ni) − *E*(BGDY-3Ni), (3)

where *E*(BGDY-2Ni) is the energy of the most stable structure with two adsorbed Ni atoms, that is, the alternate configuration of Figure 5. *E_ads_*(3rd Ni atom) in the chain configuration, which is given in Table 1, is smaller than *E_ads_*(2nd Ni atom) and similar to *E_ads_*(1st Ni atom) because the third Ni atom does not form bonds with the other two.

## 6. Adsorption of Six Ni Atoms per Cell

**Figure 8:**
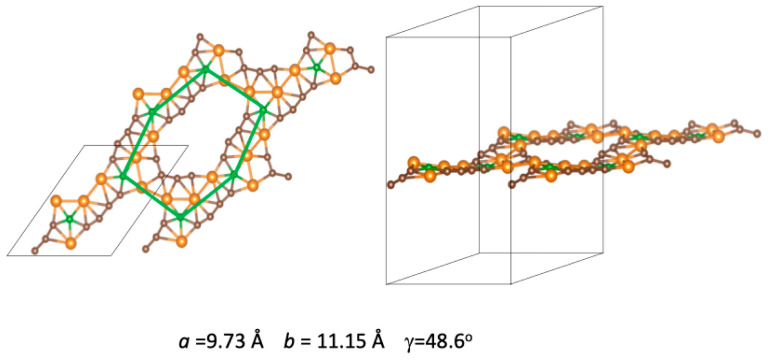
Top and side views of the most stable geometry corresponding to the functionalization of BGDY with six Ni atoms per cell. The calculations allowed for full relaxation of *a*, *b*, and γ. One deformed hexagon is sketched by drawing green straight lines connecting the B atoms.

**Figure 9:**
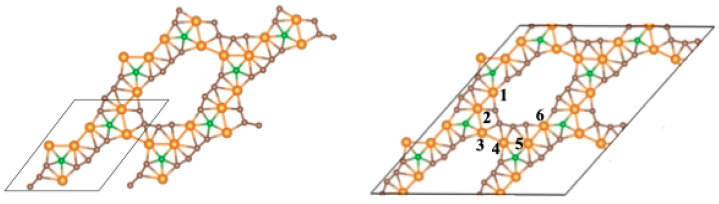
(**Left panel**) Top view of the structure predicted for BGDY functionalized with six Ni atoms per cell. Four cells are shown. (**Right panel**) structure predicted using a cell four times bigger. The two predicted structures are identical. Nickel atoms of the curved chain are labeled.

Decorating the unit cell with six Ni atoms allows for a single initial configuration, with the six Ni atoms occupying the six corners of each hexagon. The structure obtained after optimization is shown in Figure 8. The deviation of the BGDY layer from planarity is almost negligible. The in-plane distortion of the BGDY framework is sizable, with γ = 48.6° and a marked difference between *a* and *b*. The hexagonal holes of BGDY deform substantially. One distorted hexagon is sketched in Figure 8 by the plotted straight lines connecting B atoms. The distortion of the hexagonal structure is due to the formation of bonds between Ni atoms: curved chains of six Ni atoms can be noticed, and the distances between adjacent Ni atoms (labeled from 1 to 6 in Figure 9) are *d*(1-2) = 2.33 Å, *d*(2-3) = 2.43 Å, *d*(3-4) = 2.43 Å, *d*(4-5) = 2.46 Å, and *d*(5-6) = 2.37 Å. These Ni–Ni distances are lower than the nearest neighbor distances in bulk Ni and thus closer to the Ni–Ni distances expected in small Ni clusters. Each B atom, bonded to three C atoms in pristine BGDY, is now bonded to two C atoms and to three Ni atoms. The carbon chains joining the B atoms are separated into two groups: three chains are located inside the deformed hexagon delineated in Figure 8, and three chains are outside. Their locations alternate. Four near-linear carbon chains still join B atoms, while the ends of the other two carbon chains are not linked to B atoms. This structure is quite different compared to the structures predicted for the adsorption of one, two, and three Ni atoms per cell, and the deformation of the BGDY layer is much larger.

One may ask if the new predicted structure of BGDY decorated with six Ni atoms per cell depends on the size of the unit cell adopted in the calculations. The answer is provided in Figure 9, where the results for calculations with the 1 × 1 unit cell are compared to results using the 2 × 2 unit cell (four times bigger). The conclusion is that the structure does not depend on the size of the cell and indicates that this is a unique structure. In the lower panel of Figure 9, one can clearly appreciate that the Ni atoms form a network in which chains of six Ni atoms are linked through B atoms. This is relevant to interpreting the band structure in Section 7. Observation of Figure 2, Figure 5, Figure 7 and Figure 9 confirms the adaptability of the Ni atoms to different atomic environments. Ni atoms with atomic coordination between four (four neighbors) and eight (eight neighbors) are observed in those Figures.

## 7. Electronic Structure of the Ni-Doped BGDY Layers

**Figure 10:**
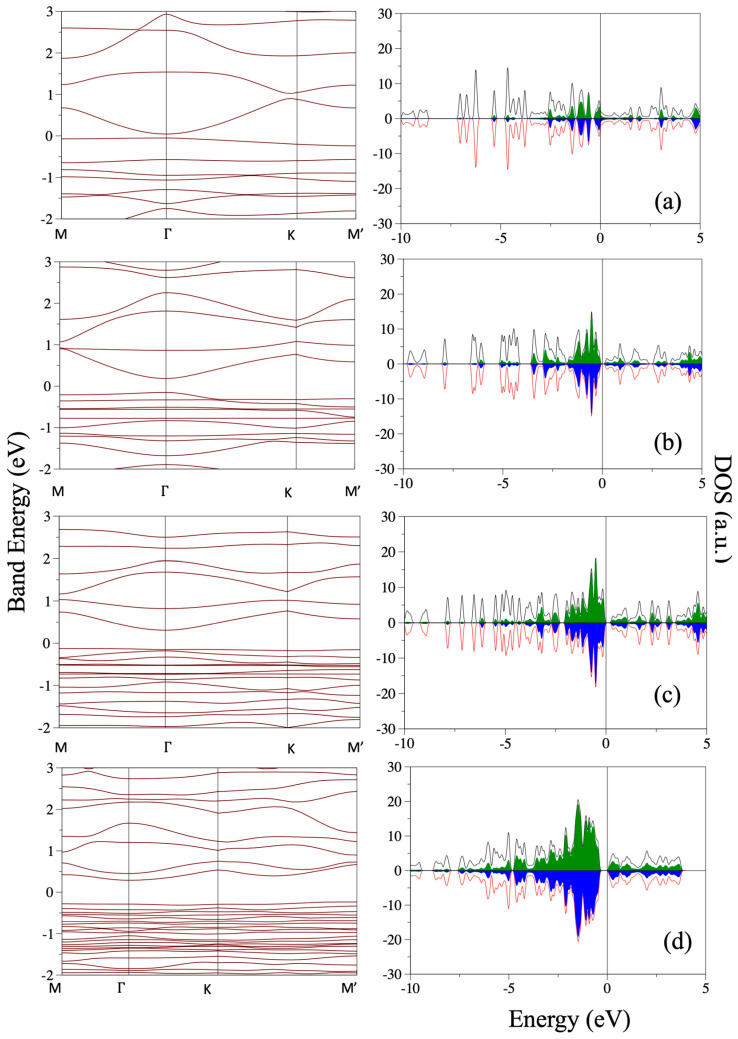
Electronic band structure (**left**) and density of states (**right**) BGDY-1Ni (**a**), BGDY-2Ni (**b**), BGDY-3Ni (**c**), and BGDY-6Ni (**d**). Black and red curves correspond, respectively, to spin-up and spin-down states (there is perfect matching between up and down bands). The green and blue filled areas are DOSs projected on the Ni atoms.

To investigate the electronic properties of the BGDY layers doped with Ni, the spin-polarized electronic band structure has been calculated. To obtain accurate results for the band structures, a 20 × 20 × 1 *k*-point mesh was used, although we discovered that a smaller 11 × 11 × 1 *k*-point mesh already led to converged results. When calculating the band structure, the lowest energy geometrical structures obtained in the previous Sections were maintained fixed. The electronic energy band structures and the corresponding densities of electronic states (DOSs) of BGDY doped with one, two, three, and six Ni atoms per cell, called here BGDY-1Ni, BGDY-2Ni, BGDY-3Ni, and BGDY-6Ni, respectively, are shown in Figure 10. The DOSs are plotted in the energy range between −10 eV and +5 eV. In all cases, the Ni-doped layers have a semiconducting character. The electronic energy band gap is direct, and it occurs at the Γ point. The band structure evolves smoothly as the number of Ni atoms per cell increases. The gap is quite narrow (0.096 eV) for BGDY-1Ni. Then, the gap increases with the number of Ni atoms: 0.337 eV in BGDY-2Ni, 0.460 eV in BGDY-3Ni, and 0.578 eV in BGDY-6Ni. The occupied electronic bands near the Fermi level have a predominant nickel *d*-like character, while the unoccupied bands present a hybridized mixed character. The occupied bands near the Fermi level are quite flat. This is due to the sizable distances between Ni atoms in BGDY-1Ni, between Ni dimers in BGDY-2Ni, and between Ni chains in BGDY-6Ni. The layer BGDY-3Ni contains both Ni dimers and Ni atoms, and the distances are also sizable between any of these. However, as the number of Ni atoms per unit cell grows, the interaction between the Ni atoms, either direct or indirect through the hybridization with C and B atoms, increases. Although this interaction is modest, it progressively enhances the stability of the material, a feature responsible for the increasing widening of the gap. The conclusion is that the width of the gap can be tailored by varying the amount of Ni doping. The gap widths between 0.4 eV and 0.6 eV for BGDY-2Ni, BGDY-3Ni, and BGDY-6Ni suggest that these materials may be promising for infrared photodetectors. The bands and the DOS reveal a perfect matching between the spin-up and spin-down bands, and the predicted magnetic moments of these materials are exactly 0 μ_B_. This is due to the hybridization of the Ni and C orbitals. In self-consistent calculations of the electronic structure of magnetic systems, the magnetic state obtained can depend on the magnetic configuration assumed at the start of the calculation. We have taken care of this fact by considering several initial magnetic configurations with non-zero magnetic moments on the atoms, and, in all these cases, the calculations converged to configurations having total magnetic moments of 0 μ_B_ per cell and also full local compensation of up and down spins, that is, zero local magnetic moments.

## 8. Structural and Thermal Stability of the BGDY-6Ni Layer

The thermal stability of the BGDY-6Ni layer material has been probed by performing ab initio DFT molecular dynamics simulations. Constant temperature simulations have been performed at three different temperatures, 500 K, 1000 K, and 1500 K, and the layer has been modeled with increasingly large, 2 × 2, 3 × 2, and 4 × 3, repetitive supercells to better accommodate possible structural transformations of the layer. Since the AIMD simulations are computationally highly demanding, the smaller 2 × 2 supercells allow us to run longer trajectories of up to 12 ps, whereas simulation times for the largest 4 × 3 supercells will be limited to 2.7 ps. A comparison of the AIMD results for the three simulation cells is presented in the Supplementary Information.

**Figure 11:**
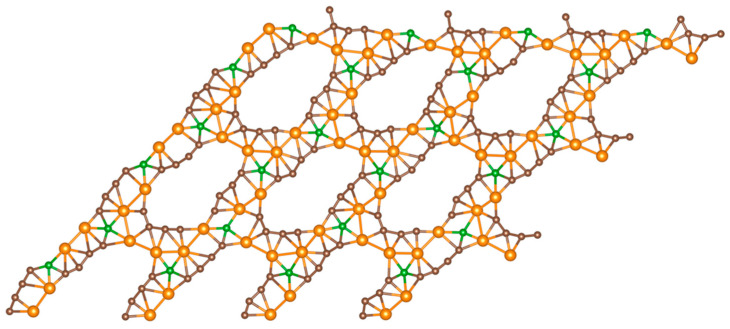
Snapshot of the BGDY-6Ni 4 × 3 supercell taken from a trajectory at 1500 K after a running time of 2.6 ps.

Visual inspection of all the AIMD trajectories shows that the atoms oscillate around the equilibrium positions. Those oscillations are faster and of larger amplitudes with increasing temperature. However, the structure of the layer is well preserved: the local restructuring of atoms leading to structural transformations was never observed, and the diffusion of atoms along the cell never happened. This indicates an extraordinarily high stability of the BGDY-6Ni layer up to temperatures of at least 1500 K. Figure 11 shows a snapshot of the dynamics for the 4 × 3 supercell at 1500 K after 2.6 ps. This structure cannot be distinguished by the eye from the most stable configuration of the layer given in Figure 8, which confirms the absence of structural transformations. Of course, in Figure 11, the atoms are slightly displaced from their corresponding equilibrium positions due to vibrations.

A more quantitative characterization of the displacements of the atoms along a trajectory is provided by the time dependence of the mean square displacement, msd, (average over all atoms in the supercell of the square of the distance between the initial position of the atom and its position at a later time). As shown in Figure S1 of the Supplementary Information, msd increases almost linearly at short times and tends to be a small constant value (different values for different temperatures) at long times, which is a signature of solid-like behavior and indicates no structural transitions, that is, the thermal stability of the layer.

**Figure 12:**
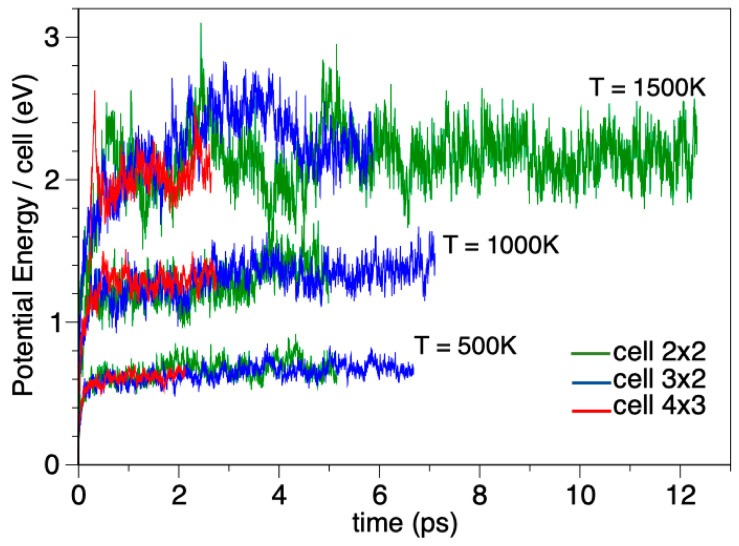
Potential energy with respect to the energy of the most stable configuration of the BGDY-6Ni layer as a function of time. Results for the 2 × 2, 3 × 2, and 4 × 3 supercells are provided for temperatures T = 500, 1000, and 1500 K.

The energetics of the layer along trajectories of increasing temperatures, T = 500, 1000, and 1500 K, have also been analyzed. Figure 12 depicts the potential energy with respect to the energy of the most stable configuration, $∆E\left(t\right),$ of the BGDY-6Ni layer as a function of time. $∆E\left(t\right)$ is defined as follows:

$$∆E\left(t\right)=E\left(MD,\;t\right)-E\left(Ground\;State\right)$$

where $E\left(Ground\;State\right)$ is the energy of the lowest energy configuration and $E\left(MD,\;t\right)$ is the potential energy of the layer evaluated along the simulated trajectories. Similar oscillations of $∆E\left(t\right)$ and the same average value at long times are obtained for the three supercells for each simulation temperature. The amplitude of the oscillations increases with increasing temperature. This energy difference, which represents the loss of stability of the layer with respect to the ground state configuration, oscillates around a constant value (a different one for each temperature). The oscillations and the constant average value of the energy are consistent with the oscillation of the atoms around the lowest energy structure and the absence of structural changes.

**Figure 13:**
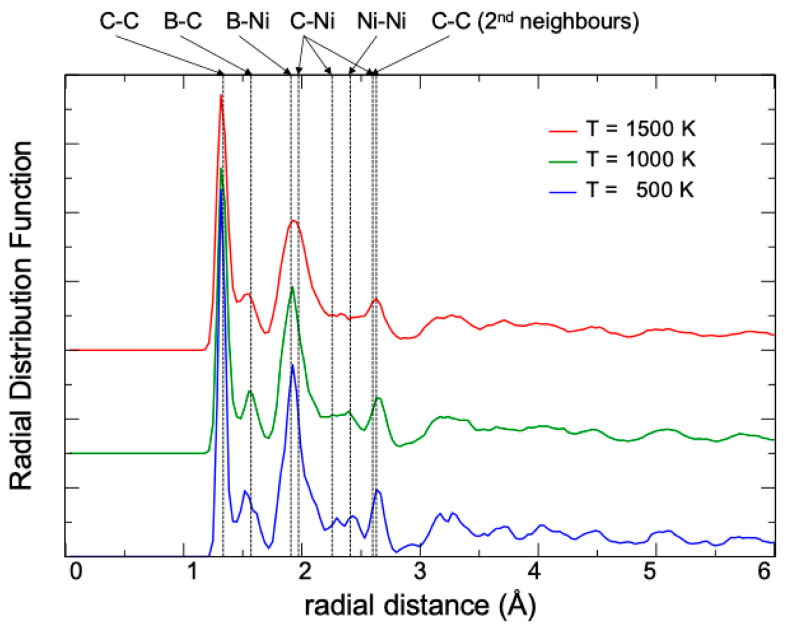
The radial distribution function of BGDY-6Ni at three different temperatures. The rdf has been evaluated from MD simulations using the 4 × 3 supercell. Vertical dashed lines correspond to the average distances between A–B atoms, as indicated in the figure, in the lowest energy configuration at T = 0 K. The graphs corresponding to the different temperatures have been vertically shifted for easier visualization.

The evolution of the structural characteristics of the layer with increasing temperature can be analyzed by plotting the radial distribution function, rdf. Figure 13 shows the rdf of the BGDY-6Ni layer for three temperatures (see also Figure S2 of the Supplementary Information). The rdf at T = 500 K shows well-defined peaks corresponding to the average distances between neighboring atoms. The peaks in the rdf show a small broadening, which is mainly due to the temperature. With increasing temperature, the peaks become just a little broader, and some of the smaller peaks are blurred out. However, the position of the peaks remains stable with increasing temperature, and there is no evidence of either structural changes or phase transformations up to 1500 K. In summary, AIMD simulations performed on large supercells up to high temperatures, 1500 K, demonstrate the extraordinary thermal stability of the BGDY-6Ni layers, which makes this novel material very attractive for technological applications.

**Figure 14:**
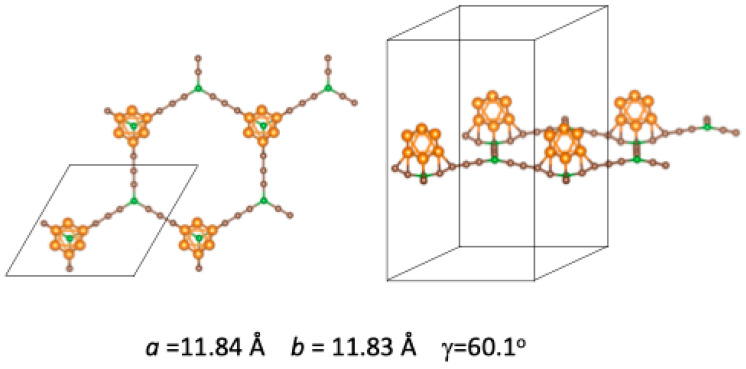
Top and side views of the most stable geometry for the functionalization of BGDY with one Ni_6_ cluster per cell.

Finally, the BGDY-6Ni decoration is compared with the adsorption of Ni_6_ clusters on BGDY. The calculated lowest energy structure of free Ni_6_ is a slightly distorted octahedron. The results of the adsorption of the Ni_6_ cluster (one cluster per cell), allowing for full optimization of *a*, *b*, and γ, are displayed in Figure 14. The cluster sits on top of a B atom of the BGDY layer, with two faces of the octahedron parallel to the layer. The structure of the cluster and the BGDY framework are practically undistorted, but a small buckling of the layer is noticed. The adsorption energy of the cluster,

E_ads_(Ni_6_) = E(BGDY) + E(Ni_6_) − E(Ni_6_@BGDY), (4)

is 4.68 eV. However, a comparison of the total energy of the configuration of the adsorbed Ni_6_ cluster, E(Ni_6_@BGDY), with the total energy of the decoration structure, E(BGDY-6Ni), of Figure 8, reveals that the decoration structure is more stable by 4.62 eV per cell. Another difference, with respect to decoration, is that the functionalization with the Ni_6_ cluster leads to a magnetic structure with a magnetic moment of 2.2 μ_B_ per cell, in contrast to 0 μ_B_ per cell obtained for the decorated structure. This is due to the three-dimensional structure of Ni_6_ and the smaller interaction with the substrate.

The results in this and previous sections reveal that the adsorption of Ni atoms and clusters induces distortions of the planarity of the BGDY layers as well as in-plane distortions. The deviations from planarity are small, although buckling is noticed in some cases. The stability gained through in-plane structural distortions arises from the formation of Ni–Ni, Ni–B, and Ni–C bonds. This is the reason why decoration with six Ni atoms per cell is so stable. The adsorption of Ni_6_ clusters minimizes the layer distortion; however, the number of bonds between Ni atoms and the atoms of the supporting layer is small.

The interaction energies between Ni atoms, measured, for instance, by the cohesive energies of the Ni bulk metal [45], 4.44 eV per atom, are large. Then, the adsorption of the Ni_6_ clusters on BGDY could be expected to be energetically competitive with the decoration configuration. However, Ni decoration is more stable. First of all, the structural rearrangement of the BGDY framework in BGDY-6Ni allows for the formation of some Ni–Ni bonds; in addition, each B atom forms bonds with three Ni atoms, and the interaction between B and Ni atoms is strong, as demonstrated by the presence of several Ni–B intermetallic compounds in the Ni–B alloy phase diagram [46]. Those effects stabilize the decoration configuration and prevent the agglomeration of the adsorbed Ni atoms into clusters.

## 9. Conclusions

Density functional calculations have been performed to investigate the interaction between Ni atoms and clusters and two-dimensional (2D) boron graphdiyne layers. The adsorption of one, two, and three Ni atoms per cell leads to in-plane distortions of the BGDY framework, and novel two-dimensional structures are predicted with the Ni atoms decorating the BGDY layer. An intriguing 2D structure, with the BGDY layer drastically distorted, is found for decoration with six Ni atoms. All the Ni-decorated BGDY layers have a semiconducting character. The width of the electronic gap can be tailored by varying the amount of Ni doping, and BGDY-2Ni, BGDY-3Ni, and BGDY-6Ni can be promising materials for infrared detectors. This work shows that computer simulation can be utilized to discover new materials by the functionalization of layered carbon materials with metal atoms. We expect that the experimental synthesis of these materials can be possible by the chemical vapor deposition of Ni on the BGDY substrate. The Ni-doped BGDY platforms may be interesting in catalysis. In particular, the BGDY-1Ni layers could have applications in single-atom catalysis. Also, it may be worth exploring the possibilities of BGDY-2Ni, BGDY-3Ni, and BGDY-6Ni as hydrogen storage materials.

## Figures and Tables

**Table 1:** Adsorption energies (in eV) of the first, second, and third Ni atoms on BGDY.

	*E_ads_* (eV)
first Ni atom	3.37
second Ni atom	4.09
third Ni atom	3.23

**Table 2:** Relative energies (in eV) of the optimized (final) structures corresponding to the three starting configurations with two Ni atoms adsorbed in adjacent (chain), alternate, and opposite positions in the hexagons. The structures are shown in Figure 5. The zero energy corresponds to the most stable structure. Also, adsorption energies (in eV) of the second Ni atom are shown.

Relative Ni Positions	Relative Energy (eV)	Adsorption Energy of Second Ni Atom (eV)
chain	0.90	3.19
alternate	0.00	4.09
opposite	0.70	3.39

**Table 3:** Relative energies (in eV per cell) of the final structures corresponding to the three starting configurations (chain, alternate, and intermediate) for the functionalization of BGDY with three Ni atoms per cell. The zero energy corresponds to the most stable structure.

Relative Ni Positions	Relative Energy (eV)	Adsorption Energy of Third Ni Atom (eV)
chain	0.00	3.23
alternate	0.93	2.30
intermediate	0.85	2.38

## Data Availability

Data are contained within the article and Supplementary Materials.

## Supplementary Materials

The following supporting information can be downloaded at https://www.mdpi.com/article/10.3390/nano14211706/s1, Figure S1. Mean square atomic displacement of the BGDY-6Ni layer as a function of time. Results for the 2 × 2, 3 × 2 and 4 × 3 supercells are provided for temperatures, T = 500, 1000 and 1500 K. Figure S2. Radial distribution function of the BGDY-6Ni layer. Results for the 2 × 2, 3 × 2 and 4 × 3 supercells are provided for temperatures, T = 500, 1000 and 1500 K. Vertical dashed lines correspond to the average distance between A-B atoms, as indicated in the figure, in the lowest energy (zero temperature) configuration. The graphs corresponding to the different temperatures have been vertically shifted for an easier visualization.
