# Supplementary material for: New Two-Dimensional Materials Obtained by Functionalization of Boron Graphdiyne Layers with Nickel
Source: Nanomaterials (Basel). 2024 Oct 25;14(21):1706. doi: 10.3390/nano14211706 (PMC11547700; doi:10.3390/nano14211706)
Supplement: Supplementary file 1 [file nanomaterials-14-01706-s001.zip › nanomaterials-3240809-supplementary.pdf]

Supplementary Information

New two-dimensional materials obtained by functionalization of boron graphdiyne layers with nickel

Estefanía Germán[1*], María J. López[1] and Julio A. Alonso[1,2]

1. Departamento de Física Teórica, Atómica y Optica, University of Valladolid, 47011 Valladolid, Spain
2. Donostia International Physics Center (DIPC), 20018 San Sebastián, Spain

*corresponding author: estefania.german@uva.es

Ab initio constant temperature molecular dynamics simulations of the BGDY-6Ni layer have been performed at three different temperatures, T=500, 1000 and 1500 K. The layer is modelled with increasingly large, 2×2, 3×2 and 4×3, repetitive supercells that contain a total of 80, 120 and 240 atoms, respectively. All the simulations started at the equilibrium configuration of the layer.

Figures S1 and S2 show the comparison of the dynamical results for the three supercells at the three investigated temperatures.

Figure S1 presents the mean square displacement, msd (the average over all atoms in the supercell of the square of the distance between the initial position of the atom and its position at a later time), of the BGDY-6Ni layer as a function of time. Similar results are obtained for the three supercells at each temperature although a somewhat smoother behaviour, that is, smaller amplitude oscillations as a function of time are observed for the larger 4×3 supercell.

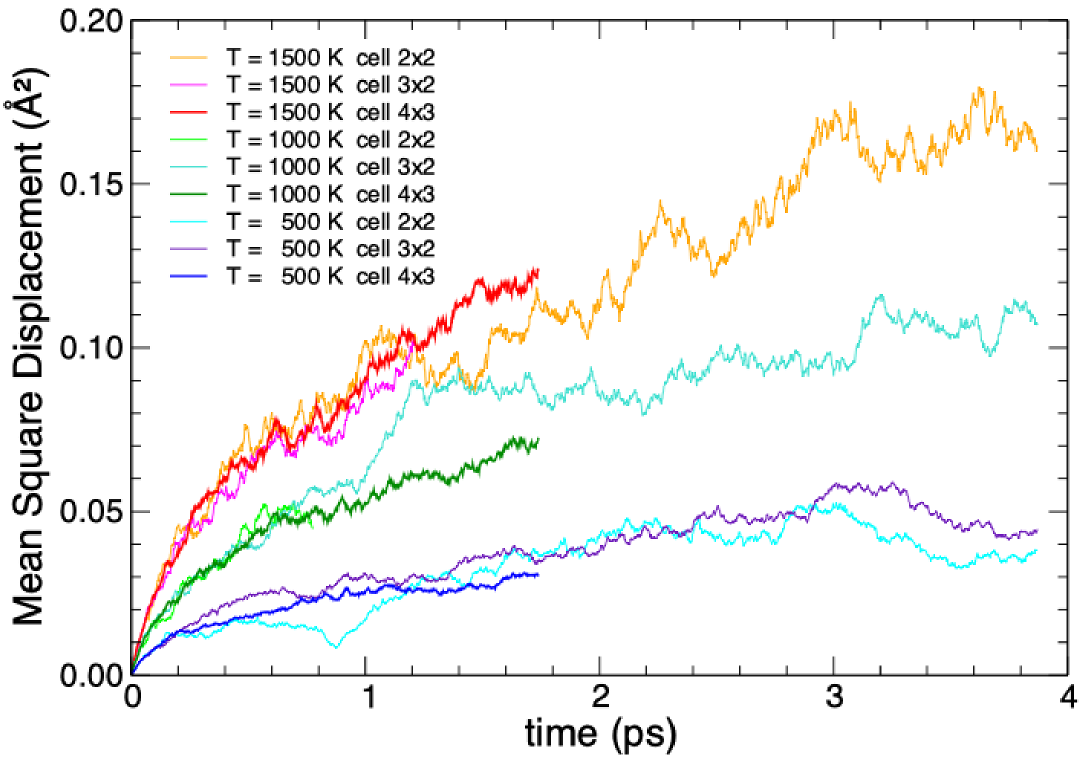

Figure S1. Mean square atomic displacement of the BGDY-6Ni layer as a function of time. Results for the 2×2, 3×2 and 4×3 supercells are provided for temperatures, T=500, 1000 and 1500 K.

Figure S2 depicts the radial distribution function of the BGDY-6Ni layer. The results of the three supercells are almost identical for each temperature.

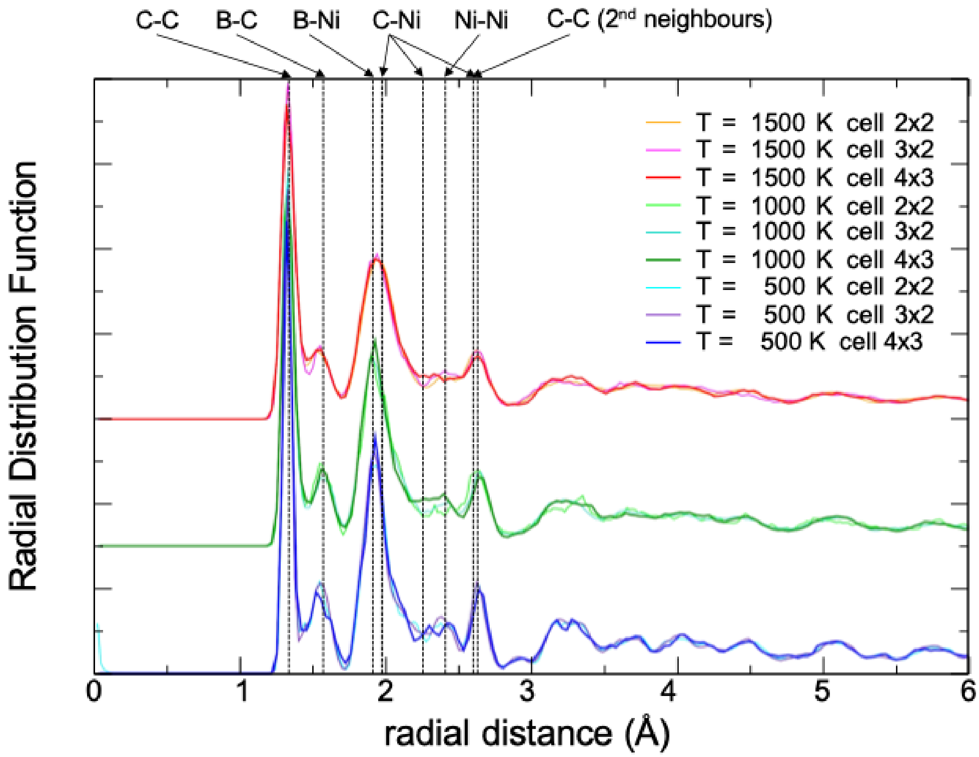

Figure S2. Radial distribution function of the BGDY-6Ni layer. Results for the 2×2, 3×2 and 4×3 supercells are provided for temperatures, T=500, 1000 and 1500 K. Vertical dashed lines correspond to the average distance between A-B atoms, as indicated in the figure, in the lowest energy (zero temperature) configuration. The graphs corresponding to the different temperatures have been vertically shifted for an easier visualization.

In summary we have shown that the simulations with the three supercells provide consistent results and therefore one can obtain a better insight from the simulations by combining long simulation times for small cells with shorter simulation times for the larger cells.
